# Modality, utilization and mortality in pediatric ESKD: a registry-based study

**DOI:** 10.3389/fneph.2026.1930091

**Published:** 2026-10-26

**Authors:** Subodh J. Saggi, Anil Mongia, Angelika C. Gruessner, Mary Mallappallil, Moro Salifu

**Affiliations:** 1 Department of Medicine, Division of Nephrology, SUNY Downstate Health Sciences University, Brooklyn, NY, United States; 2 Division of Pediatric Nephrology, SUNY Downstate Health Sciences University, Brooklyn, NY, United States; 3 Department of Medicine, Division of Nephrology, NYC Health + Hospitals/Kings County, Brooklyn, NY, United States

**Keywords:** dialysis, end-stage kidney disease, kidney transplantation, pediatric patients, peritoneal dialysis, renal disease

## Abstract

**Introduction:**

Pediatric end-stage renal disease (ESKD) is rare but clinically complex. This study evaluated modality utilization, transplant access, and mortality among pediatric ESKD patients in the IPRO Network registry.

**Methods:**

We performed a retrospective registry-based analysis of 1,628 pediatric ESKD patients from four U.S. regions between 2018 and 2024. Descriptive statistics summarized patient characteristics and treatment patterns. Competing risk models were used to assess time to modality transition, transplant listing, transplantation, and survival.

**Results:**

Pediatric patients represented 0.65% of all ESKD cases in the network. Peritoneal dialysis (PD) was the most common initial modality in children aged 0–2 years (72%), compared with 24% in those aged 15–17 years. Overall, 46% started hemodialysis (HD), 31% PD, and 23% were preemptively listed for transplantation. Transitions from HD to PD were uncommon. Transplant listing occurred in 63% of patients by 1 year and 81% by 3 years; transplantation occurred in 22% and 53%, respectively. One-, two-, and three-year mortality rates were 3.4%, 5.4%, and 6.6%, with the highest mortality rate in children aged 0–2 years (10% at 3 years).

**Discussion:**

PD is the preferred initial modality in very young children, while older children more often start hemodialysis. Overall survival and transplant access were favorable, but infants and toddlers remained the most vulnerable subgroup.

**Conclusion:**

Pediatric ESKD accounts for a small but distinct share of the ESKD population. Early transplant access and good short-term survival were observed, but outcomes remain poorest in the youngest children.

## Introduction

End-stage renal disease (ESKD) in children (<18 years old) is the final, irreversible stage of chronic kidney disease (CKD), characterized by permanent loss of kidney function. Although ESKD is less common in children than adults, it presents unique medical, developmental, and psychosocial challenges (1–5).

The causes of ESKD in children differ from those in adults, with congenital and hereditary disorders predominating, particularly CAKUT, cystic kidney disease, Alport syndrome, and acquired glomerular diseases such as FSGS, HUS, and lupus nephritis.

Regardless of the cause, progressive nephron loss leads to uremia, metabolic acidosis, hypertension, anemia, and growth impairment. Eventually, the glomerular filtration rate (GFR) falls below 15 mL/min/1.73 m², signaling the need for dialysis or transplantation.

In contrast to adults, children with ESKD face major developmental and psychosocial challenges, including impaired growth, delayed puberty, cognitive effects of uremia, psychological stress, and substantial family burden from dialysis care and frequent medical visits. Those issues require special attention in this age group.

Hemodialysis (HD), peritoneal dialysis (PD), and kidney transplantation are the 3 main kidney replacement therapy (KRT) options which are used in children. Each modality has distinct advantages, limitations, and age-appropriate considerations. Selecting an appropriate initial modality and ensuring timely transitions is critical to optimizing both survival and quality of life in this vulnerable population. Consequently, choices in KRT modalities in this population remain a challenge. National data from the USRDS (2, 6) and from NAPRTCS (7) regarding modality choices in pediatric ESKD patients are not granular enough to conduct meaningful statistical analysis based on disease categories, gender and age. The “Island Peer Review Organization (IPRO)’ (8) is a nonprofit healthcare quality improvement organization (QIO) that contracts with Centers for Medicare & Medicaid Services (CMS) to oversee quality of dialysis care in multiple U.S. states and territories. IPRO database has more granular data describing the movements between different treatment modalities and hence we used this database to analyze trends in KRT modality utilization and mortality among pediatric ESKD patients, in the New England, New York, South Atlantic and Ohio River regions. The reporting is mandatory. Our goal is to highlight variations in KRT care across different care settings that have potential for mitigation.

## Methods

This retrospective analysis included information from 1,628 pediatric patients with ESKD in the Island Peer Review Organization (IPRO) ESKD Network. The IPRO Network is a mandatory federally designated program that supports high-quality, patient-centered care for individuals with end-stage kidney disease across multiple U.S. regions (8).

Four regional networks were included in the analysis: New England (Connecticut, Maine, Massachusetts, New Hampshire, Rhode Island, and Vermont), New York (New York State), South Atlantic (Georgia, North Carolina, and South Carolina), and the Ohio River Valley (Indiana, Kentucky, and Ohio).

The analytic file was built from all patient registrations, admissions, events, listings, transplant, and death records reported to the IPRO networks. All pediatric patients (under 18 years) who entered one of these networks between 2018 and 2024 were included and followed through the study period, with the database closed on December 31, 2024. **Figure 1** illustrates the transitions between the different modalities.

**Figure 1:**
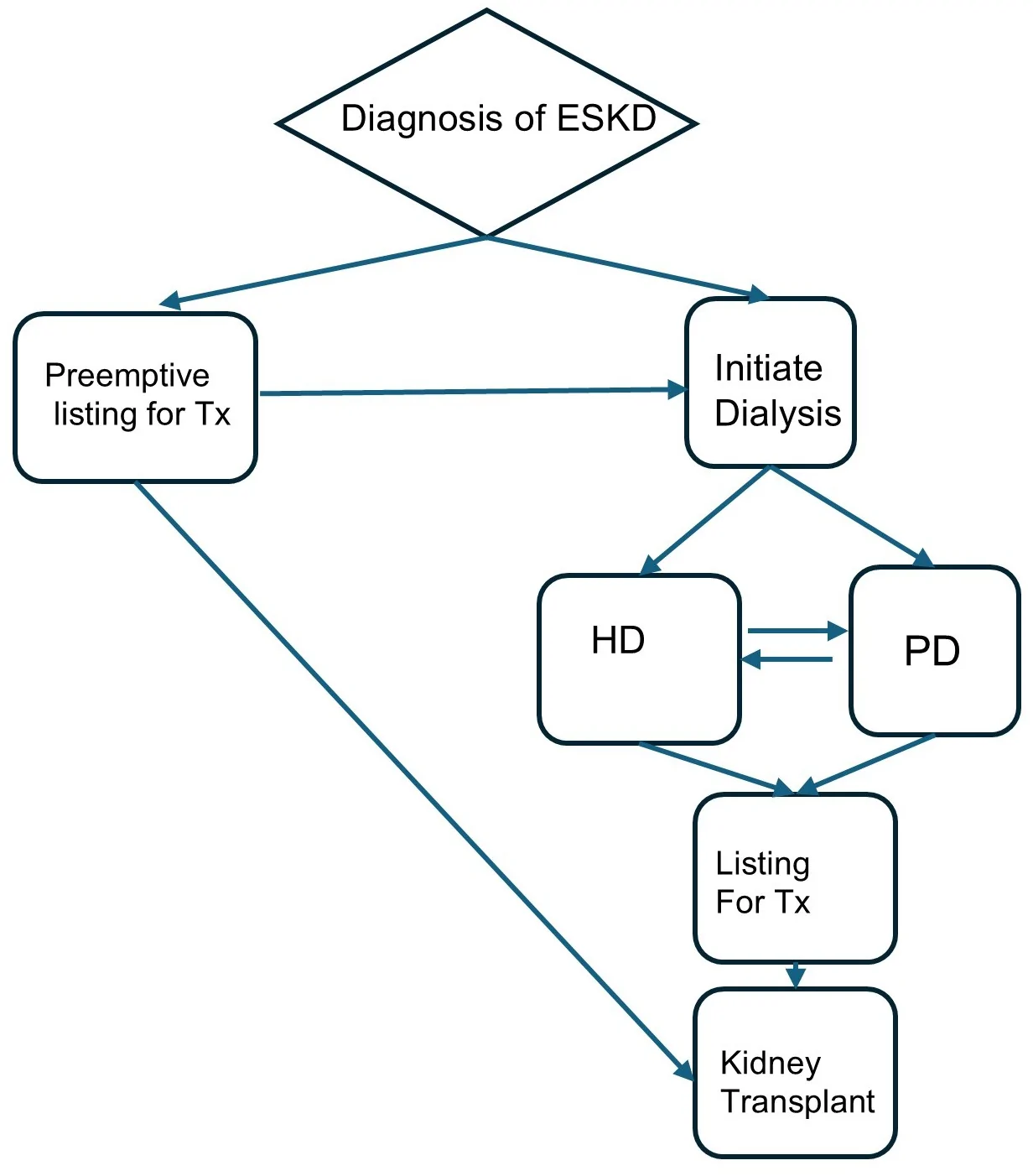
Flowchart of treatment modalities for 1,628 children with ESKD across 4 IPRO networks listed between 2018 and 2024.

To assess impact across the pediatric age spectrum, patients were stratified into 4 groups based on WHO/CDC recommendation: 0-2years (infants), 3–7 years (preschool/early childhood), 8–14 years (adolescent), and 15–17 years (older adolescents).

Comprehensive descriptive analyses were conducted using medians (range) for continuous variables and frequencies (percentages) for categorical variables. Analyses focused on baseline treatment modality, transitions between HD and PD, from ESKD diagnosis to time of listing and time to kidney transplantation, and mortality after transplantation. Because the multiple treatment modalities represented competing risks, a competing risks model was used to estimate event rates over time for initiation of PD, listing and kidney transplantation. For patients on HD, the competing events for PD initiation were kidney transplantation or death. For the time to listing and for kidney transplantation, death before the event was the competing risk.

For analyses of patients on PD, censoring mechanisms varied depending on the question: For the analysis of time on PD, transition to transplantation, death or transition back to HD was counted as end on PD. For PD failure, transition to transplantation was censored, whereas return to hemodialysis or death was counted as a failure. Overall patient survival was assessed from the time of dialysis initiation. We developed a time-dependent Cox regression model, beginning at dialysis initiation and adjusted for age, gender, and network, to compare survival between children on dialysis and children after kidney transplantation.

All tests were two-tailed and performed at a significance level of 0.05. Analyses were conducted using SAS 9.4 software (SAS Institute, Cary, NC). This study was exempt from review by the SUNY Downstate Health Sciences University Institutional Review Board.

## Results

### Patient population

Among 252,075 individuals with end-stage kidney disease (ESKD) included in the analysis, only 0.65% were pediatric patients (**Table 1**) with newly diagnosed ESKD. Very few children were registered at rural clinics; the majority were treated at large urban centers, reflecting the ongoing centralization of pediatric nephrology services in the 4 networks.

**Table 1:** Basic characteristics of 1,628 pediatric patients with ESKD in 4 IPRO networks listed between 2018 and 2024.

Region	New England	New York	South Atlantic	Ohio River Valley	Total
# of Patients (%)	245 (15)	366 (23)	544 (33)	473 (29)	1,628 (100)
Initial Age (yrs)	12 (0 - 17)	13 (0 - 17)	12 (0 - 17)	11 (0 - 17)	12 (0 - 17)
Male (%)	155 (63)	218 (60)	313 (58)	273 (58)	959 (59)
Race (%)					
White	127 (52)	111 (30)	188 (35)	270 (57)	696 (43)
Black	19 (8)	81 (22)	181 (33)	89 (19)	370 (23)
Hispanic	55 (22)	93 (26)	90 (16)	35 (8)	273 (17)
Asian	1 (0)	12 (3)	3 (1)	2 (0)	18 (1)
Other	21 (9)	29 (8)	20 (4)	19 (4)	89 (5)
Unknown	22 (9)	40 (11)	62 (11)	58 (12)	182 (11)
Rural (%)	3 (1)	0 (0)	1 (0)	0 (0)	4 (0)
Baseline Treatment (%)					
Hemodialysis	116 (47)	190 (52)	242 (45)	209 (44)	757 (46)
PD	54 (22)	70 (19)	203 (37)	170 (36)	497 (31)
Preempt. Listing	75 (31)	104 (28)	99 (18)	94 (20)	372 (23)
Transplant	0 (0)	2 (1)	0 (0)	0 (0)	2 (0)
Mortality after dialysis (%)					
1 year	6.8%	2.2%	3.4%	2.9%	3.4%
2 Year	11.8%	3.7%	3.4%	6.1%	5.4%
3 -Year	17.3%	3.7%	3.4%	7.9%	6.6%

### Patient characteristics

The age and gender distribution (**Figure 2A**) was bimodal, with one peak at ages 0–2 years and a second at 8–17 years. At the time of first listing in the IPRO registry, a significant gender difference was observed, with a male predominance that was especially pronounced in the youngest and oldest age group.

**Figure 2:**
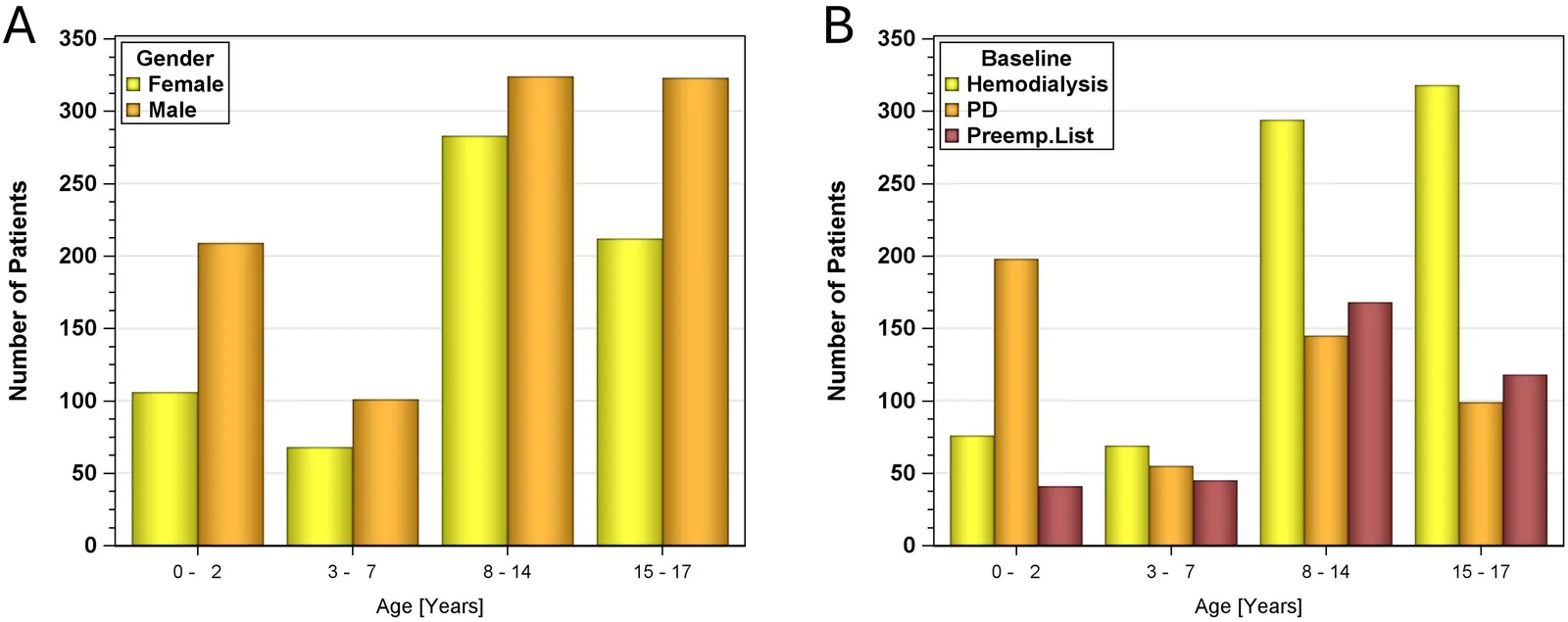
Age distribution by gender **(A)** and baseline treatment modalities by age category **(B)** for 1,628 children with ESKD in 4 IPRO networks listed between 2018 and 2024.

The racial distribution differed significantly across the four networks. White children constituted the majority in the Ohio River Valley and the New England networks, whereas Black children were more prevalent in the South Atlantic and New York networks. The New York network had also the highest proportion of Hispanic children (**Table 1**).

### Dialysis modality

Because of vascular access limitations in children 2 years of age or younger, peritoneal dialysis (PD) was the most frequent initial treatment modality (**Figure 2B**). Among children aged 0–2 years, 72% initiated dialysis with PD, compared with 24% among those aged 15–17 years (*p* < 0.0001). The cumulative incidence of children starting PD varied across age groups (**Figure 3A**) and IPRO networks (**Figure 3B**), with the South Atlantic and Ohio River regions showing the highest initiation rates (>45%), whereas the New York (27%) and New England (32%) networks had significantly lower rates.

**Figure 3:**
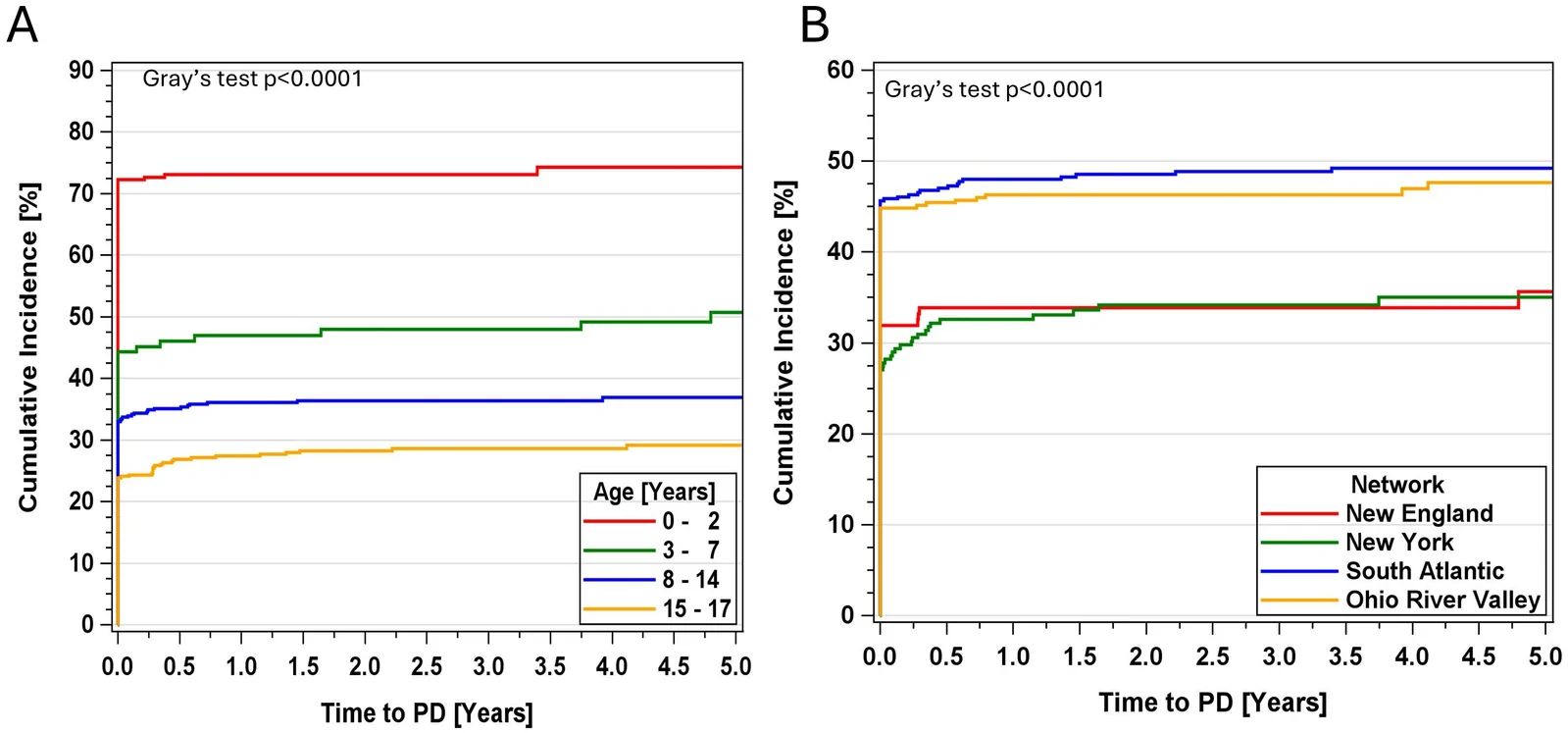
Cumulative incidence of time to initiate PD by age **(A)** and network **(B)** among children with ESKD across 4 IPRO networks listed between 2018 and 2024. Competing events before initiation of PD were kidney transplantation or death. Children who started with PD are plotted at time 0.

Race significantly influenced the initial treatment modality. White children had a markedly higher rate of preemptive listing (31%) than Black (14%) or Hispanic children (24%), and they overwhelmingly began treatment on hemodialysis.

Among children who initially started on hemodialysis (HD), overall, only additional 5% were transferred to PD during the first year and overall, 7% by year 3. Age and gender did not influence later transitions to PD; however, patients within the New York network converted to PD earlier than those in other regions (*p* <0.0001) (**Figure 3B**).

During the study period, only 5 children used Home HD: two infants, two adolescents, and one older adolescent. Because the number was so low, these children are summarized together with the HD children.

### Course on peritoneal dialysis

Children initiating PD or transitioning from HD to PD generally remained on PD for a limited period before transitioning to kidney transplantation (**Figure 4A**). Less commonly were removal due to technique failure or death. At 3 years, overall, only 16% of patients had continued PD. PD duration varied by age: at 1 year after PD initiation, 76% of children aged 0–2 years and less than 60% of those aged 15–17 years remained on PD.

**Figure 4:**
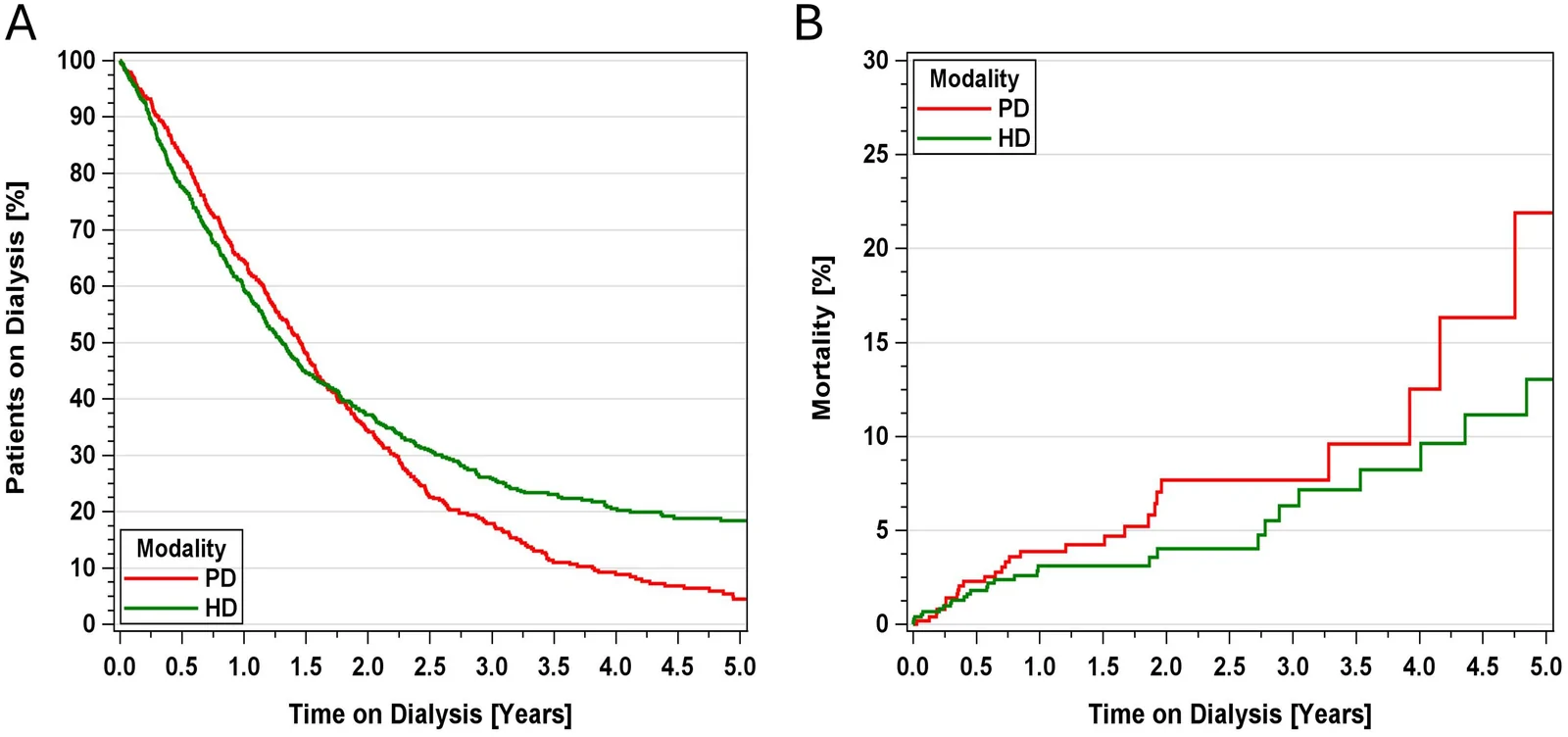
Comparison of time on dialysis **(A)** and mortality after start of dialysis **(B)** between children on HD and those on PD among children with ESKD across 4 IPRO networks listed between 2018 and 2024.

Children in the New England and New York network stayed longer on PD compared to children in the Ohio River Valley and South Atlantic network (p=0.04).

Technique failure was most frequent in the youngest age group (*0–2 years*, 15% at 1 year), followed by 8% in ages 3–14 years and 11% in ages 15–17 years.

Mortality in children while being on PD was low. The 1-year mortality rate was 4%, limited to the youngest patients (0–2 years). Older age groups showed no increase in mortality.

In comparison to HD, children on PD stayed a shorter time on dialysis (**Figure 4A**) but mortality for children on HD was only slightly higher when they stayed for a longer time on dialysis. (**Figure 4B**).

### Transplant listing and outcome

Children were promptly listed for kidney transplantation: the cumulative incidence of children listed preemptively was 35% and overall increased to 63% within 1 year and 81% within 3 years after registry enrollment. Listing occurred more slowly in children aged 0–2 years (*p* < 0.001), whereas there was no significant difference among older age groups. At year 1, listing rates were 30% for those age 0-2, but over 70% for the older children (**Figure 5A**).

**Figure 5:**
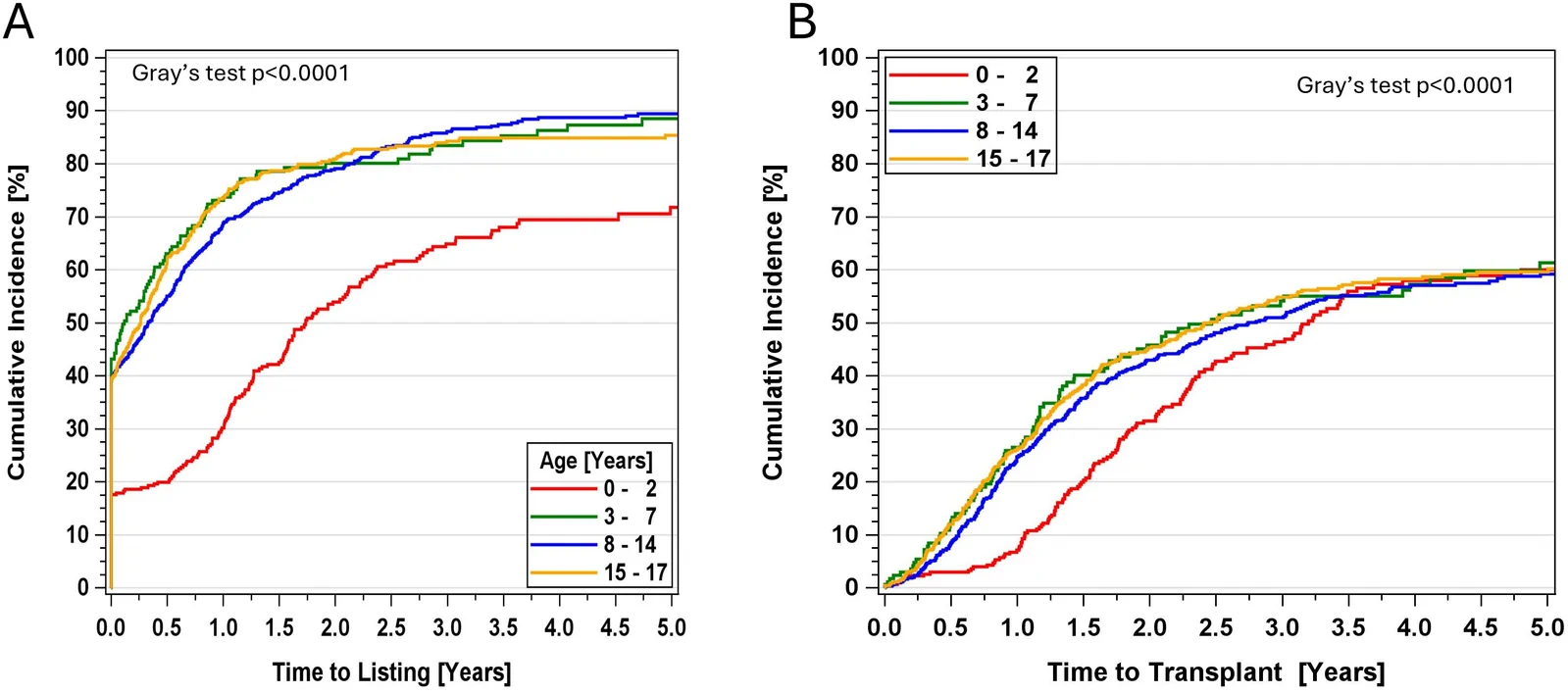
Cumulative incidence of time from initial diagnosis to listing (Preemptively listed children are plotted at time 0) **(A)** and time to kidney transplantation **(B)** by age among children with ESKD across 4 IPRO networks, 2018–2024. The competing event was death before the event of interest.

The cumulative incidence of transplantation independent of modality was 22% at 1 year and 53% at 3 years (**Figure 5B**). Neither gender nor network was associated with time to transplantation The initial delay in transplantation observed among the youngest children (0–2 years) was attributable to later listing; among those aged 3–17 years, no difference in transplantation rate was observed (*p* > 0.7).

Of the 793 children who received a kidney transplant, most kidneys (71%) came from deceased donors, 17% from living related donors, and 12% from living unrelated donors.

### Survival outcomes

Overall survival following dialysis initiation (HD or PD) was excellent. One-, 2-, and 3-year mortality rates were 3.4%, 5.4%, and 6.6%, respectively. The highest mortality occurred in the youngest age group (0–2 years), reaching 10% at 3 years (**Figure 6**). No differences in survival were noted for the children between the ages of 3 and 17. Survival did not differ by gender.

**Figure 6:**
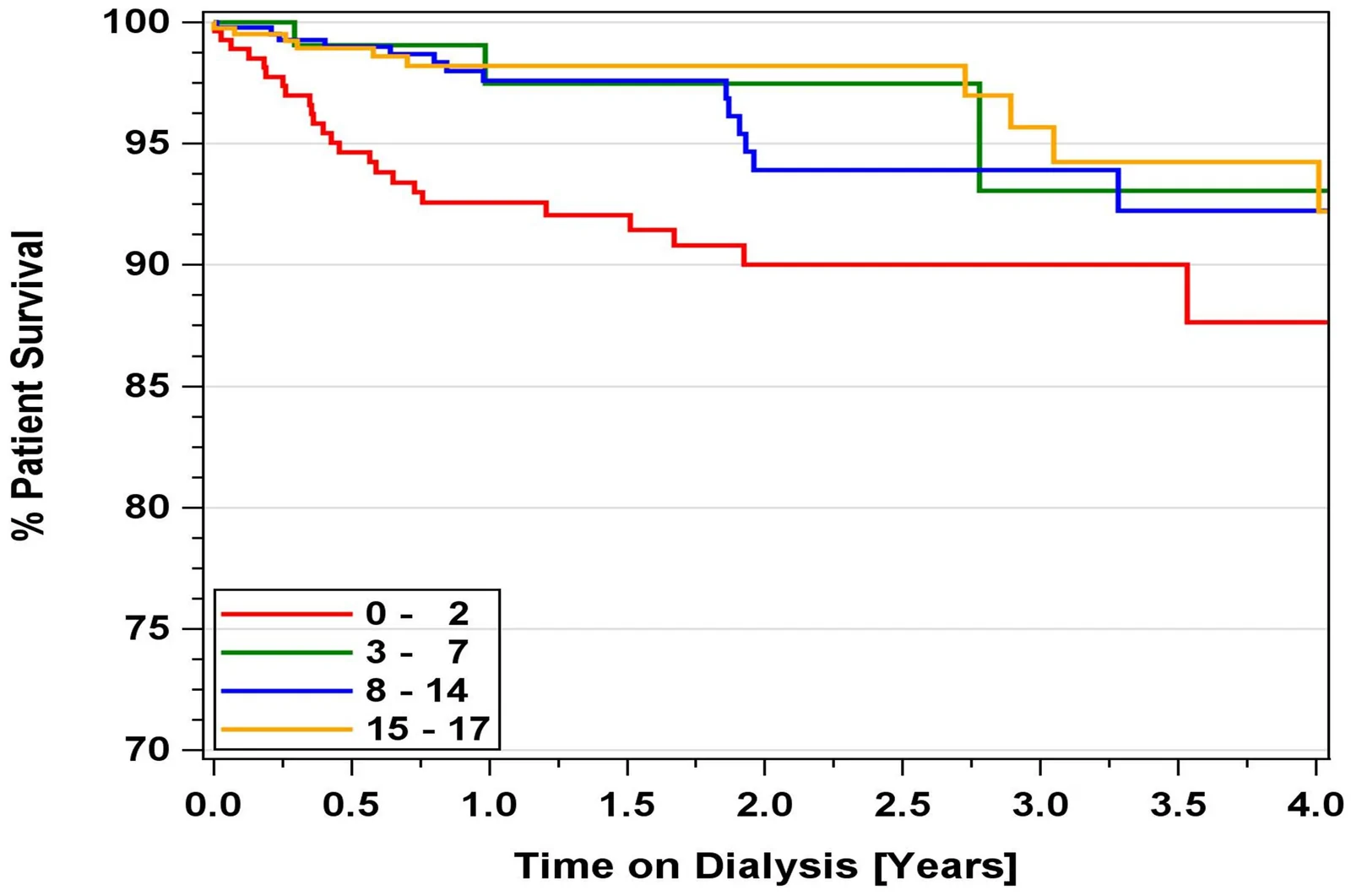
Overall patient survival after start of dialysis by age, independent of dialysis modality, among children with ESKD across 4 IPRO networks listed between 2018 and 2024.

Short-term mortality did not vary significantly among networks (*p* = 0.07), but long-term survival was lower for children enrolled in the New England network than in New York (*p* = 0.02).

Among children who received a kidney transplant after being on dialysis, one- and three-year patient survival was 100% and 99%, respectively.

Only six children died after receiving a kidney transplant during the analyzed period. Children who received a transplant had significantly higher survival compared to children still waiting for a transplant (p < 0.0001). Comparable one- and three-year survival among children still waiting on dialysis was 95% and 93%.

In a multivariable time-dependent analysis of the risk of dying, transplantation was associated with a significantly lower risk than remaining on dialysis (HR 0.04, 95% CI 0.02–0.09). Influential factors were age and network. Children in the 0–2-year category had twice the risk of dying compared with all other children (HR 2.20, 95% CI 1.15–4.23). The risk of dying was significantly increased in the New England network compared with the other three networks (HR 3.04, 95% CI 1.33–6.94). No impact of gender on survival was found (*p* = 0.51).

## Discussion

Here, we report data from 1,628 pediatric ESKD patients across New England, New York, South Atlantic, and Ohio River regions collected through the IPRO Network registry, focusing on modality utilization and mortality within the pediatric ESKD population. We further compared these outcomes with those from other major renal registries, including NAPRTCS, USRDS, and the 2024 KIDIGO Guideline Key Points for Pediatricians (9).

Drawing on such data from over 252,000 individuals in the IPRO registry with ESKD from period 2018-2024, pediatric patients represented only a small but clinically distinct population (0.65%) of total ESKD burden which is in agreement with prior estimates that children constitute fewer than 1% of the total ESKD load (1, 10).

According to the literature, the bimodal age distribution was based on the underlying etiologies - with congenital anomalies of the kidney and urinary tract (CAKUT) and cystic kidney diseases predominating in younger children, and glomerulopathies and systemic disorders in older children and adolescents. CAKUT remains the leading cause of chronic kidney disease in children (3, 11), particularly in those under five years of age, and its higher prevalence in boys is consistent with the known gender-linked predisposition of conditions such as posterior urethral valves (PUV) and vesicoureteral reflux (VUR) (12–14).

We noticed a predominance of peritoneal dialysis (PD) as the initial modality in young children, particularly those aged 0–2 years (72%), which compares with other registry data from NAPRTC (8) and European studies (15, 16). Vascular access for hemodialysis remains technically challenging in small children, and PD offers several other practical advantages in infants and toddlers, including hemodynamic stability, ease of home administration, and preservation of residual renal function (17–23). Home HD was reported in only 5 children, while PD remained the preferred modality even in older children.

The regional variation in PD initiation rates observed in this study - with the South Atlantic and Ohio River networks showing notably higher rates (>45%) than New York and New England (32%) likely reflects a combination of differences in patients transition to kidney transplantation, sociocultural background, center experience, and regional clinical practice which was also detected in several other studies (24–26). These disparities highlight the importance of standardizing access to PD across IPRO networks and warrant further investigation (21, 23).

We found that the transition from one dialysis modality to another was low. The transition rates after start of hemodialysis (HD) to PD (5% at one year, 7% at three years), and the absence of age- or gender-based predictors of transition, suggest that the initial modality choice tends to be maintained over time. This has implications for pre-dialysis counseling and the importance of informed consent regarding modality selection at the outset of renal replacement therapy (27–29). The earlier conversion to PD observed in the New York network may again reflect greater institutional expertise or patient-family preference for home-based therapy (21).

Technical failure on PD was most frequent in the youngest age group (15% at one year), which is consistent with published literature identifying infant age, low body weight, and additional co-morbidities. Frequent complications remain the leading causes of therapy failure in pediatric PD, and efforts to reduce infection rates are critical in this vulnerable cohort (30, 31).

Mortality during dialysis was low overall, with 1-, 2-, and 3-year rates of 3.4%, 5.4%, and 6.6%, respectively. These figures compare favorably with historical pediatric ESKD mortality data and likely reflect overall improvements in dialysis management (32). The concentration of mortality in the youngest age group (10% at 3 years) is a well-recognized fact attributable to the higher burden of comorbid condition, and the physiological challenges of renal replacement in infants (33, 34). The trend toward higher mortality among infants on PD - though not statistically warrants continued monitoring and may achieve significance with larger sample sizes or longer follow-up.

The high rates of transplant listing (57% at one year, 77% at three years) and transplantation (22% at one year, 53% at three years) are encouraging, reflecting an appropriate emphasis of kidney transplantation as the optimal long-term renal replacement therapy in children (35, 36). The delayed listing and transplantation observed in the youngest age group may reflect the clinical and logistical challenges of transplanting very small recipients, including minimum weight thresholds, technical surgical demands, and recipient vascular considerations (37, 38). Notably, once children reached three years of age, no significant difference in transplantation rates was observed across older age groups, suggesting an equitable distribution of transplant access among school-age children and adolescents. Noticeable was the better survival for children who had received a kidney transplant compared to children who were still on dialysis waiting.

The lack of significant survival differences by gender is consistent with most pediatric ESKD cohort studies (39). The lower long-term survival observed in the New England network compared with New York (p = 0.02) is an intriguing finding that merits further study. Possible explanations may include differences in center volume, patient complexity, access to subspecialty care, or socioeconomic and demographic factors (40).

Strength of study: To our knowledge, this study represents one of the first analyses of pediatric data derived from an IPRO organization, highlighting an area that has been relatively understudied. Although highly published, NAPRTCS and USRDS registries are primarily research and surveillance oriented while IPRO’s Networks are operationally and quality-improvement focused. In contrast to the IPRO networks were reporting is mandatory for all patients, a major limitation of NAPRTC registry is that participation is voluntary and not geographically comprehensive. On the other hand, USRDS relies heavily on Medicare claims data, which means analyses are limited to the ESKD population with Medicare fee-for-service insurance coverage, potentially excluding younger privately insured patients.

Several limitations of this study should be acknowledged. Information regarding the underlying diseases that led to ESRD was not available. The registry data along with retrospective design and missing underlying disease precludes causal inference, and unmeasured confounders may influence the observed associations. Only 4 out of the 1,628 children were treated in a rural setting, which prevented us from comparing outcomes between those supervised in urban versus rural centers. Furthermore, the small absolute number of pediatric patients, while representing the largest available cohort, limits statistical power in subgroup analyses, as evidenced by several borderline p-values. In addition, due to the unavailability of underlying disease information, we could not compare mortality rates between cases with CAKUT diagnosed prenatally (via antenatal ultrasound) and those diagnosed postnatally. Data on the weight and height at the time of initiating dialysis and at the final follow-up was not available, hindering our ability to assess the impact of malnutrition on mortality, a factor that could significantly influence outcomes.

In conclusion, this analysis provides a comprehensive characterization of pediatric ESKD in 4 large networks. It reaffirms the primacy of PD in young children, documents meaningful regional variation in dialysis practice, and underscores the concentration of morbidity and mortality among infants. These findings should motivate quality improvement initiatives, pre-dialysis counseling practices, and future prospective research aimed at optimizing outcomes in one of the most medically complex and vulnerable patient populations in nephrology. It also shows the excellent transition from dialysis modalities to kidney transplantation.

## Implications from real-world IPRO data

Peritoneal dialysis (PD) is often favored over hemodialysis in pediatric patients because it provides gentler, continuous therapy, avoids the challenges of vascular access, preserves residual kidney function, and can be performed at home with less disruption to growth, development, and school attendance.

These findings suggest that medical centers should allocate greater resources to PD training to improve outcomes and consider broader training in minimally invasive PD catheter placement.

Pediatric patients with ESKD should also be listed for kidney transplantation and transplanted as early as possible, as transplantation offers the best opportunity for survival, normal growth, neurocognitive development, and improved quality of life, whereas prolonged dialysis may result in lasting harm. Accordingly, academic medical centers should invest in specialized training for pediatric transplant nephrologists and surgeons.

Such strategies may improve pediatric outcomes and quality of life while expediting waitlisting and transplantation.

## Data Availability

The datasets presented in this article are not readily available because IPRO is the owner of the data set and the information is restricted. Requests to access the datasets should be directed to “Svetlana Lyulkin”.
